# Eradication of CD48-positive tumors by selectively enhanced YTS cells harnessing the lncRNA NeST

**DOI:** 10.1016/j.isci.2023.107284

**Published:** 2023-07-05

**Authors:** Rebecca Kotzur, Natan Stein, Shira Kahlon, Orit Berhani, Batya Isaacson, Ofer Mandelboim

**Affiliations:** 1 The Lautenberg Center for Immunology and Cancer Research, the Hebrew University, Medical School Hadassah Ein Karem, Israel, Jerusalem

**Keywords:** Cellular therapy, Components of the immune system, Cancer

## Abstract

Natural killer (NK) cells are currently used in clinical trials to treat tumors. However, such therapies still suffer from problems such as donor variability, reproducibility, and more, which prevent a wider use of NK cells therapeutics. Here we show a potential immunotherapy combining NK cell-mediated tumor eradiation and long non-coding (lnc) RNAs. We overexpressed the interferon (IFN) γ secretion-enhancing lncRNA nettoie Salmonella pas Theiler’s (NeST) in the NK cell-like cell line YTS. YTS cells express the co-stimulatory receptor 2B4 whose main ligand is CD48. On YTS cells, 2B4 functions by direct activation. We showed that NeST overexpression in YTS cells resulted in increased IFNγ release upon interaction with CD48 (selectively enhanced (se)YTS cells). Following irradiation, the seYTS cells lost proliferation capacity but were still able to maintain their killing and IFNγ secretion capacities. Finally, we demonstrated that irradiated seYTS inhibit tumor growth *in vivo*. Thus, we propose seYTS cells as off-the-shelve therapy for CD48-expressing tumors.

## Highlights

• YTS, an NK tumor line, as off-the-shelf therapy for hematological malignancies
• The lncRNA NeST can be used to selectively enhance the secretion of IFNγ by YTS
• These modified seYTS cells mediated CD48-positive tumor killing via IFNγ secretion
• CD48-negative cells are spared

## Graphical abstract

**Figure:**
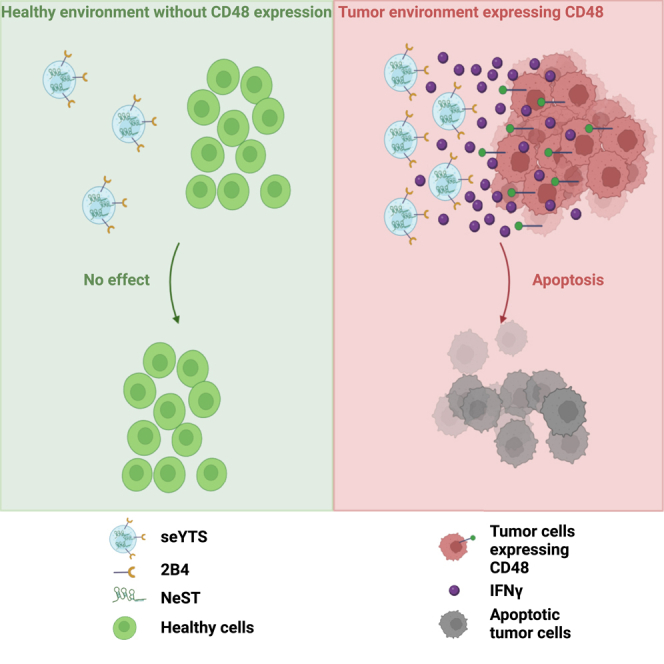

## Introduction

Immunotherapy is a novel form of cancer treatment utilizing human immune cells to fight cancer cells. The main aim behind his approach is to specifically kill cancer cells while sparing healthy and functional cells of the patient. Several different immunotherapy strategies such as monoclonal antibodies, bispecific antibodies, checkpoint inhibition, and immune cell modification (such as T and natural killer [NK] cells) are currently used.^1^

Here we focus on NK cells, due to their tumor killing potential and the already promising NK tumor therapy data generated in clinical trials around the world. NK cells used for immunotherapy can be derived from different sources including peripheral blood, umbilical cord blood, induced pluripotent stem cells (iPSCs), and clonal NK cell-like cell lines obtained from NK cell malignancies.^1^ The most used NK cell-like cell line is NK-92 which express most NK cell activating receptors.^2^^,^^3^ NK-92 cells require the addition of recombinant human interleukin-2 (IL-2) for maintenance and function, drastically shortening their lifespan and functionality *in vivo*.^3^ Also, due to their lack of inhibitory NK cell receptors, they are highly activated and can cause collateral damage to the patient apart from killing the targeted tumor cells.

YTS cells were derived from a male patient suffering from acute lymphoblastic lymphoma and express CD56^dim^ NK cell-like features.^4^^,^^5^^,^^6^ Interestingly and advantageously, they do not require addition of exogenous human IL-2 to survive and thrive in culture.^7^ YTS cells display NK cell cytotoxicity *in vitro*, but not via antibody-dependent cell-mediated cytotoxicity (ADCC) since they do not express the Fc receptor CD16.^5^ YTS express only a limited number of NK cell receptors. They do not express any functional inhibitory NK cells receptor and express very few activating ones.^4^ One of them is the co-activating receptor 2B4.^8^^,^^9^

2B4 is a surface molecule found both on murine and human NK cells and a subset of T cells. On NK cells 2B4 functions as co-stimulatory receptor; i.e., it cannot mediate cytotoxicity, on its own.^10^ It has one main binding partner, CD48.^8^^,^^11^ CD48 is expressed on a variety of hematopoietic cells, both benign and malignant.^12^^,^^13^^,^^14^ The detection of its soluble form in the peripheral blood was suggested as a potential disease marker, due to its increased expression during inflammation.^15^ CD48 binds to other receptors, one being CD2,^16^ albeit the binding affinity between CD48 and 2B4 was shown to be 6- to 9-fold stronger than the interaction between CD48 and CD2,^17^ indicating a higher physiological relevance of this interaction leading to activation of the lytic properties of NK cells.

Although, CD48 is expressed on a variety of hematopoietic cells, upregulated expression is seen in a few hematological malignancies, for example, multiple myeloma and glioma.^15^^,^^18^^,^^19^ A major study also connected the elevated expression of CD48 with a poor prognosis for glioma patients,^18^ albeit the overexpression of CD48 makes these tumor cells an optimized target for NK cell cytotoxicity.^20^

In a novel approach to optimization of immune cells for immunotherapy, this study aimed to explore the potential use of long non-coding (lnc) RNA expression in YTS cells for tumor therapy. The history of lncRNAs is relatively short due to the belief that non-coding parts of the human genome are junk and would have no function since they would never be transcribed into proteins.^21^^,^^22^^,^^23^ But due to modern sequencing and genetic interaction analysis methods, lncRNAs have been found to be regulators of gene expression within the cells.^24^^,^^25^^,^^26^ lncRNAs have been identified as being implicated in cancer development via interaction with gene regulators directly without being translated into proteins and thereby controlling cancer growth and progression if expressed inappropriately.^27^^,^^28^^,^^29^

For our immunotherapy approach we focused on the lncRNA NeST (nettoie Salmonella pas Theiler’s), or also known as IFNG-AS1 and TMEVPG1, which was initially discovered as an explanation for differential response to murine Theiler’s virus and was identified to be encoded downstream of the IFNG locus and hence suspected and later proven to influence the expression and secretion of IFNγ positively.^30^^,^^31^^,^^32^ Therefore, expression of NeST was shown to be followed by enhanced secretion of IFNγ.^30^^,^^33^ The human ortholog of this lncRNA is expressed in CD4^+^ and CD8^+^ T cells, and NK cells and their overexpression are linked to several inflammatory diseases like rheumatoid arthritis (RA), Hashimoto’s Thyroiditis (HT), and Sjogren’s syndrome.^28^^,^^34^^,^^35^^,^^36^

In this study, we aim to combine the current knowledge and create an immunotherapy utilizing the cytolytic capabilities of the NK cell-like cell line YTS, harnessing the 2B4-CD48-binding-mediated activation axis, and amplifying its effects by introducing the lncRNA as a positive regulator of IFNγ expression.

## Results

### Generation of selectively enhanced YTS cells and identification of target cells

**Figure 1:**
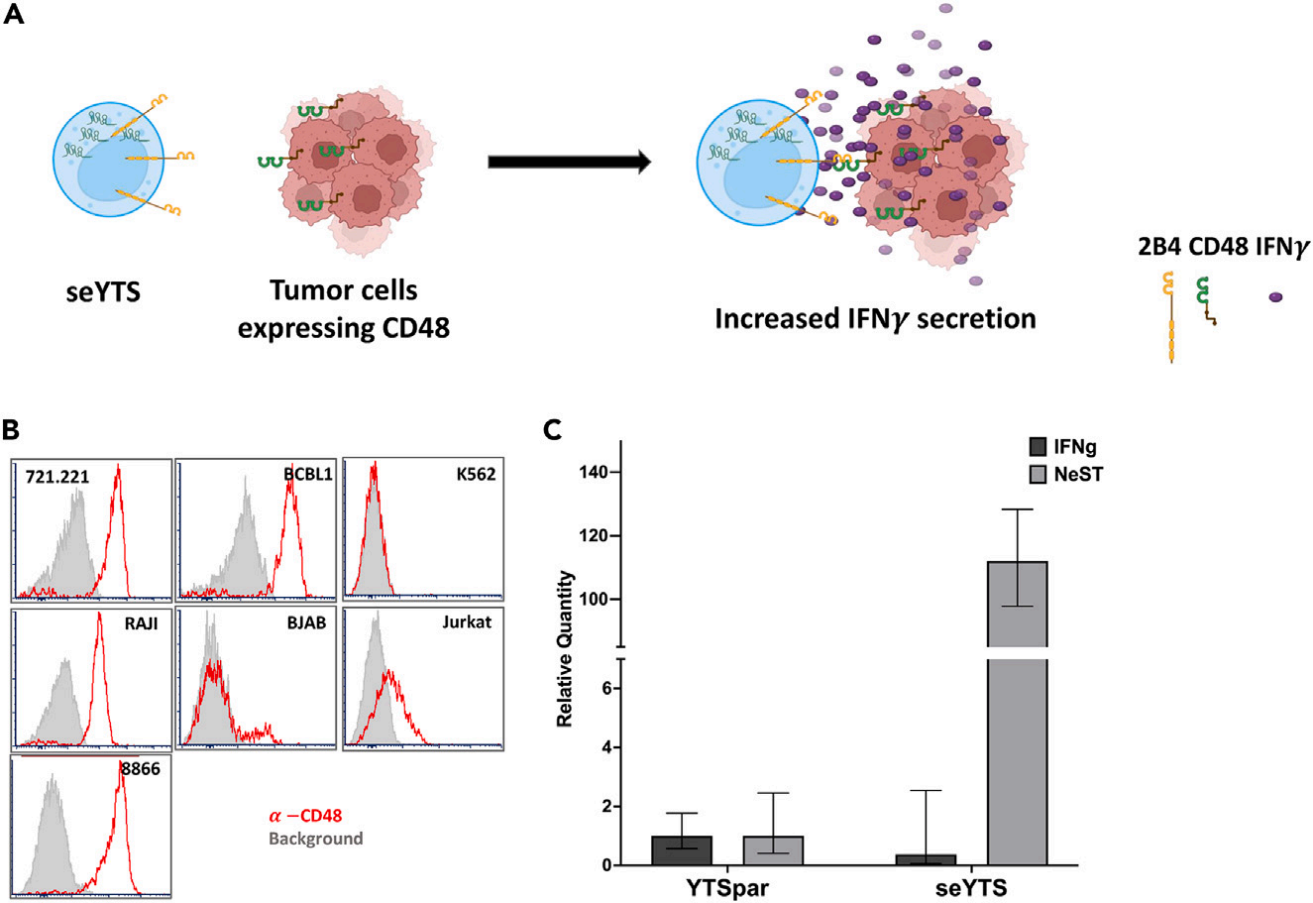
Creation of YTS overexpressing NeST and a model to test the functionality *in vitro* (A) Scheme describing the proposed mechanism of selectively enhanced (se)YTS cells activation by CD48-positive tumor cells. (B) FACS-screening for CD48 expression in liquid tumor cell lines (721.221, BCBCL1, K562, BJAB, Jurkat, RAJI, 8866), histograms gray = isotype-control background, red = anti-CD48 antibody staining. (C) 1 out of at least 3 representative experiments over a period of 1 year, RT-PCR for quantification of relative expression of human NeST and IFNG in YTS parental (YTSpar) and seYTS, GAPDH as control, N = 4, with N being technical repeats in this experiment, Data represented as mean ± SEM, medium gray = NeST, dark gray = IFNG.

The main goal of this study is to create an immunotherapeutic cell line, targeting CD48-expressing tumor cells. To achieve this aim, we generated a transgenic cell line based on the NK cell-like cell line YTS and overexpressed the lncRNA NeST (named seYTS). The idea behind this project is to use irradiated seYTS cells for the killing and IFNγ secretion following incubation with CD48-positive tumor cells (Figure 1A).

We initially wanted to identify CD48-positive lymphoid tumor cell lines. Fluorescence-activated cell sorting (FACS) staining with CD48 antibodies showed 721.221, a human B-lymphoblastoid cell line, BCBL1, a B cell lymphoma cell line, RAJI, a lymphoblast cell line, and RPMI 8866, a B-lymphoid cell line, as cells positive for CD48 expression, Jurkat, a T-lymphoid cell line, and BJAB, a lymphoma cell line, as slightly positive, and K562, a lymphoblast cell line, as CD48-negative cell line (Figure 1B). For future experiments 721.221 and BCBL1 cells were selected as positive target cells, whereas K562 cells were selected as negative controls. Next, YTS cells were modified via lentiviral transduction with the dsRed plasmid containing the NeST isoform described in Stein et al. 2019 (37). After transduction and GFP selection, NeST expression was verified via RT-PCR for NeST, GAPDH, and IFNG. The RT-PCR showed stable overexpression of NeST in the seYTS cells compared to the parental YTS, while IFNG expression remains at baseline levels (Figure 1C). These experiments were performed at various time points after the initial transduction to verify the stable long-time incorporation of the construct.

### Characterization of activation potential and off-target effects

**Figure 2:**
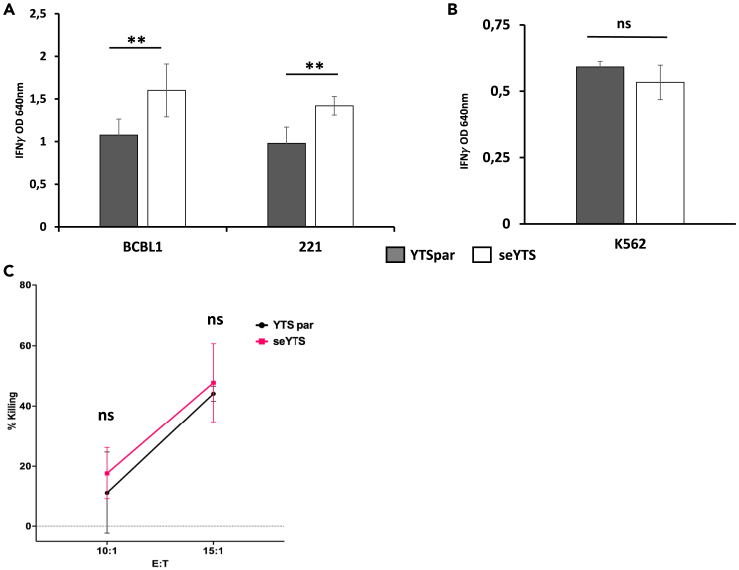
Assessment of activation of seYTS against CD48pos cancer cell lines (A and B) Activation assay determining IFNγ secretion after incubation of YTS parental (YTSpar) (gray) and seYTS (white) at an E:T of 0.5:1 for 48 h, normalized to the basic IFNγ secretion via an untreated control, with (A) CD48^pos^ cell lines BCBCL1 and 721.221 and (B) CD48^neg^ cell line K562, 1 out of 3 representative experiments, N = 3, with N being technical repeats in this experiment, Data represented as mean ± SEM, ∗∗ = p < 0.01, two-tailed ANOVA and Student’s t test performed. (C) Killing assay assessing the cytotoxic capacities of YTS parental (black) and seYTS (pink) against CD48^pos^ cell line 721.221, 1 out of 3 representative experiments, N = 3, with N being technical repeats in this experiment, Data represented as mean ± SEM, ns = non-significant, two-tailed Student’s t test and ANOVA performed.

To assess the activation and IFNγ secretion following interaction with CD48-positive target cells, the seYTS cells were incubated with the CD48-expressing target cell lines BCBL1 and 721.221 for 48 h. The cells indeed showed an elevated secretion of IFNγ measured via ELISA after the incubation compared to the parental YTS cells, after being normalized toward the basic secretion of the YTS cells of an untreated control (Figure 2A). In contrast, the CD48-negative cell line K562 did not trigger the activation of the YTS cells and therefore did not lead to IFNγ increase (Figure 2B).

Killing assay estimating the ability of the seYTS cells to eliminate the CD48-positive tumor cells illuminated that the seYTS cells do not kill 721.221 cells more efficiently than the YTS parental control (Figure 2C).

### Effect of irradiation on the functionality of seYTS cells

**Figure 3:**
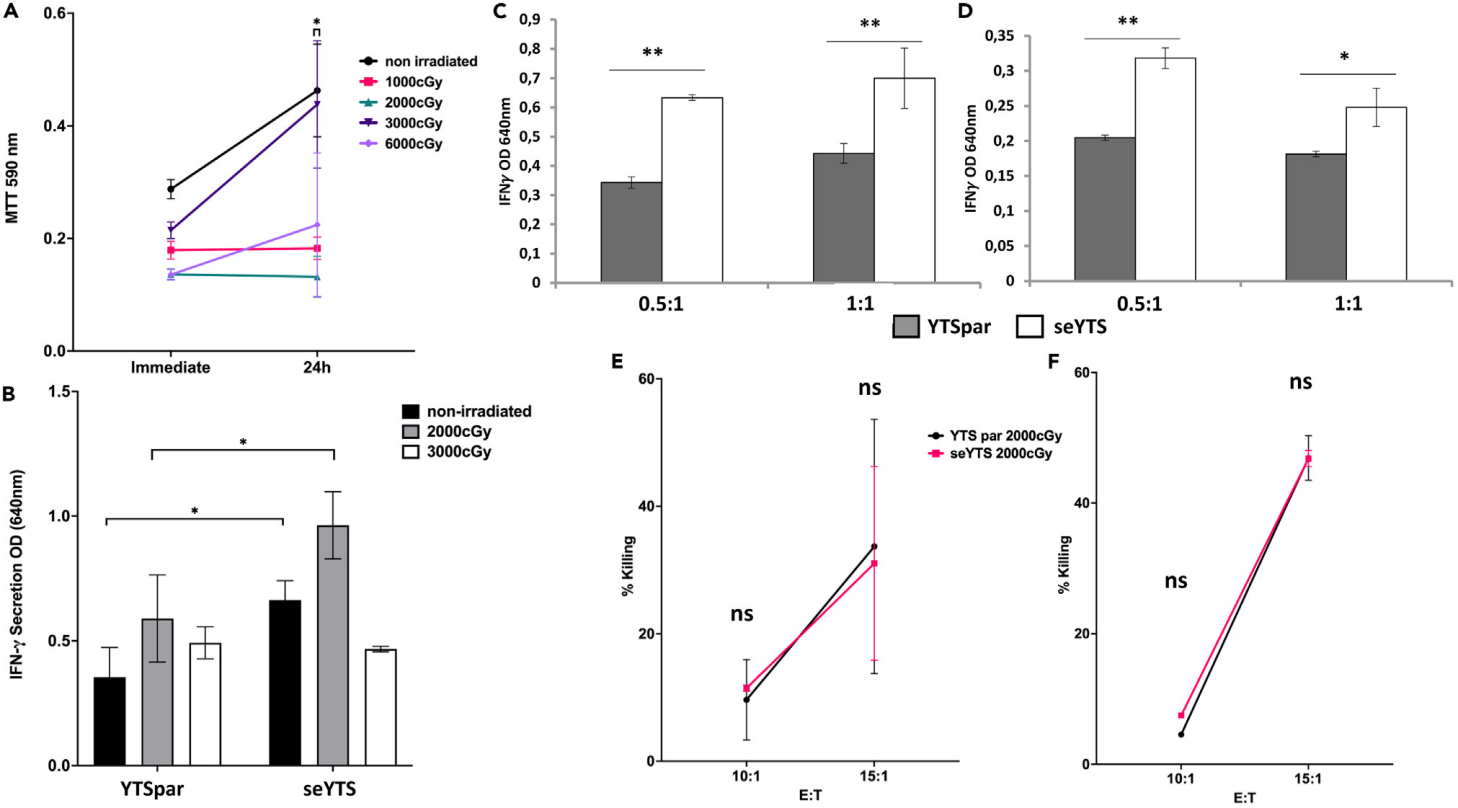
Functionality of seYTS after irradiation (A) Assessment of proliferation capacity of seYTS cells after irradiation with 1,000 (pink), 2,000 (green), 3,000 (dark violet), and 6,000 (bright violet) cGy compared to non-irradiated seYTS (black) in MTT assay over the course of 24 h, ∗ = p < 0.06, compared to non-irradiated YTS cells, 1 out of 2 representative experiments, N = 3, with N being technical repeats in this experiment, Data represented as mean ± SEM, two-tailed Student’s t test and ANOVA performed. (B) Activation with YTS parental (YTSpar) and seYTS irradiated at 2,000 (light gray) and 3,000 (white) cGy, compared to non-irradiated (black) cells incubated for 48 h at E:T 0.5:1 with BCBL1 cells, normalized to the basic IFNγ secretion via an untreated control,∗ = p < 0.06, 1 out of 2 representative experiments, N = 3, with N being technical repeats in this experiment, Data represented as mean ± SEM, two-tailed Student’s t test and ANOVA performed. (C and D) Activation assay assessing activation after incubation of YTS parental (gray) and seYTS (white), after irradiation with 2,000 cGy, for 48 h with (C) CD48^pos^ cell lines BCBL1 and (D) 721.221 at E:Ts 0.5:1 and 1:1, 1 out of 3 representative experiments, normalized to the basic IFNγ secretion via an untreated control, ∗ = p < 0.06, ∗∗ = p < 0.01, N = 3, with N being technical repeats in this experiment, Data represented as mean ± SEM, two-tailed Student’s t test and ANOVA performed. (E and F) Killing capacity after irradiation with 2,000 cGy at E:Ts 10:1 and 15:1 with YTS parental (black) and seYTS (pink) against (E) BCBL1 and (F) 721.221, 1 out of 3 representative experiments, N = 3, with N being technical repeats in this experiment, Data represented as mean ± SEM, ns = non-significant, two-tailed Student’s t test and ANOVA performed.

Since the aim of this study is to generate a new immunotherapy tool for cancer treatment and because the seYTS cells are tumor derived, the cells will need to be irradiated to prevent further replication within the patients after application. Therefore, the seYTS cells were irradiated before the next activation assay to assess their functionality after the procedure and to evaluate their proliferative capacities after irradiation. Using an MTT assay we observed that cells irradiated at 2,000, 3,000 and 6,000 cGy did not proliferate while non-irradiated cells and cells irradiated at 1,000 cGy still proliferated (Figure 3A). We then incubated the irradiated YTS cells at two doses with BCBL1 and determined that following irradiation at 2,000 cGy the YTS cells were still able to secrete IFNγ (Figure 3B). Next, we incubated BCBL1 and 721.221 cells with the irradiated YTSpar and seYTS cells at effector to target ratios of 0.5:1 and 1:1, respectively, and observed increased secretion of IFNγ following incubation with the seYTS cells (Figures 3C and 3D, respectively). The killing capacity of the 2,000-cGy-irradiated YTS cells was also not altered when compared to the YTS parental cells following incubation with BCBL1 or with 721.221 cells (Figures 3E and 3F, respectively).

### Irradiation is required before *in vivo* introduction of seYTS cells to eradicate CD48-positive tumors

**Figure 4:**
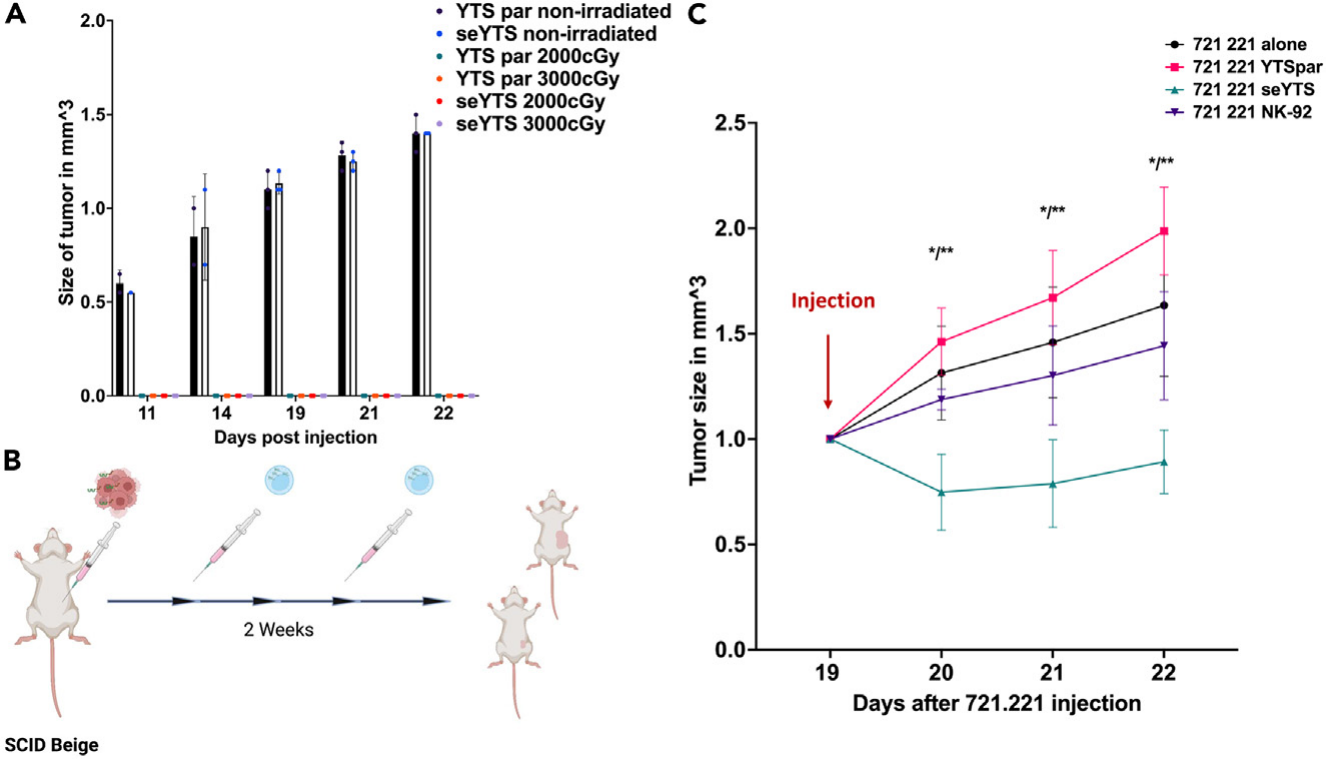
*In vivo* model assessing safety and efficiency of seYTS cells in SCID beige mice (A) Assessment of safety of the YTS cells: non-irradiated YTS parental and seYTS induce tumor growth from day 11 after injection into SCID beige mice compared to the 2,000 and 3,000 cGy irradiated YTS cells injected, N = 3, with N being individual mice, Data represented as mean ± SEM. (B) Scheme describing the *in vivo* model, 721.221 tumor cell injection into SCID beige mice, injections of immune cells (PBS, YTS parental, seYTS and NK-92 cells) at detection of palpable tumors and weekly injections at ratio 1:5 until human endpoint. (C) Efficiency of immune cells, PBS (black), YTS parental (red), seYTS (green), and NK-92 cells (violet) after injection into mice, N = 3, with N being individual mice, Data represented as mean ± SEM, ∗ = p < 0.06, ∗∗ = p < 0.01, significance of seYTS treatment compared to PBS, YTSpar and NK-92 control, 1 out of 3 representative repeats, two-tailed Student’s t test and ANOVA performed, “Injection” marks time point of treatment.

To assess the safety of the seYTS *in vivo*, parental YTS and seYTS were injected subcutaneously (s.c.) into the left flanks of severe combined immunodeficient (SCID) beige mice, deficient of T, B, and NK cells, after the cells had been irradiated at 2,000 or 3,000 cGy. The irradiated parental YTS and seYTS cells did not form tumors at all, whereas the non-irradiated parental YTS and seYTS did form significant tumors (Figure 4A). Therefore, the YTS cells should be irradiated before their application into future patients.

To assess the effect of the seYTS cells on CD48-positive tumor cells *in vivo*, mice were s.c. injected in the left flank with 1.5 × 10^7^ 721.221 cells and after establishment of palpable tumors (approximately 10–14 days after injection). After the establishment of tumors, PBS, and irradiated-at-2000 cGy YTS parental, NK-92 and the seYTS cells were injected intravenously (i.v.) in a ratio 1:5 of YTS to 721.221 cells. After the first injection, the mice received another booster with the same number of cells i.v. about a week later, if by that time point the control mice did not reach the humane endpoint (the planned experimental procedure is described in Figure 4B). *In vivo* tumor measurement showed that the seYTS cells most efficiently inhibited tumor growth compared to treatment with NK-92 cells (Figure 4C). In mice treated with YTS parental, tumors reached an even higher volume compared to tumors of mice treated with the PBS control.

## Discussion

In this study we used the NK cell-like cell line YTS and stably modified it to express the lncRNA NeST. This lncRNA positively influences the IFNγ secretion upon activation of NK cells^33^^,^^37^ and, as we show here, also enhances the secretion of IFNγ from the seYTS cells, despite the cells’ endogenous low expression of NeST.^37^ This supports the findings from Stein et al. that showed the activation of YTS overexpressing NeST and induced increased secretion of IFNγ via application of various inflammatory molecules like IL-2, IL-12, IL-15, and IL-18, as well as LPS.^37^ Since YTS cells express only one main activating receptor, 2B4, this therapy is specially designed to target CD48-positive tumors. Indeed, when we used the CD48-positive target cells 721.221 and BCBL1 cells and incubated them with the seYTS cells, increases in IFNγ secretion were observed even after irradiation, while no change was observed using the CD48-negative cell line K562. Thus, expression of NeST does not raise the activation threshold of the cells, and IFNγ secretion is enhanced only when interacting with CD48-positive tumors.

2B4 functions as co-stimulatory receptor on human primary NK cells and can thus function by its own. In contrast, 2B4 on YTS cells functions as a direct killing receptor. Therefore, seYTS are ideal for treating CD48-positive tumors. We also show that the killing activity of seYTS resembles that of parental YTS cells and is still maintained following irradiation. Thus, when seYTS cells will be injected into patients, they will not only secrete more IFNγ but will also be able to kill the targets cells.

IFNγ at the tumor microenvironment is able to interfere with tumor growth either directly by inducing apoptosis and reduction of cell proliferation or by further recruitment of adaptive immune cells.^38^^,^^39^ Additionally, recent discoveries connected the appearance of the IFNγ receptor and the activation of the IFNγR pathway with the activity and efficiency of CAR-T cell-mediated immunotherapy in solid tumors, but not in liquid ones.^40^^,^^41^^,^^42^^,^^43^

Lastly, we used irradiated seYTS to treat CD48-positive tumors after they were already established. The seYTS cells injected i.v. were able to interact with the tumors and to inhibit tumor growth presumably via IFNγ secretion. Despite the previous results indicating no increased killing potential, they were superior compared to the already tested and clinically used NK cell-like cells NK-92 cells. This is indicating an influence of the accumulation of the CD48-positive tumor cells in the tumors on the cytotoxic effects mediated by IFNγ stressing the importance of the *in vivo* study to evaluate the efficiency of the treatment. Although this work *in vivo* was performed in an immune-deficient SCID mouse model, we hope that our findings are transferable to immunocompetent patients. Since this is a murine model treated with human NK cell-like cells and, in addition, we are using a human cell line, the experiments cannot be performed in immune-competent mice as both cells will be rejected via allogeneic immune response. In human patients, we would not anticipate this reaction since it has been shown that especially NK cells are transferable between individuals without causing a rejection reaction or graft-versus-host disease (GvHD).^44^^,^^45^ Additionally, this model lets us introduce the 721.221 tumors efficiently since the mice do not have a competent immune response to counteract the tumor growth. Also, this would not be a problem in patients. Although healthy cells also express CD48 on their surface, we suspect that the expression of CD48 by these cells does not reach a threshold to efficiently recruit the seYTS and elicit a sufficient activation to harm these cells in contrast to a CD48-positive tumor.

The new immunotherapy approach we present here is exploiting a new cell molecule, lncRNAs, whereas most current NK cell-related therapies utilize CARs to target tumor cells. Our approach is utilizing already existing pathways in the cells. NeST has been identified to be binding to WDR5, a part of the H3 lysine 4 methytransferase complex, modifying the IFNG locus to enhance the expression of IFNγ.^33^ Additionally, NMD3 was detected as a potential interaction partner. This protein belongs to the ribosomal export complex. The modified seYTS cells will be activated only when encountering CD48-positive tumors, preventing unspecific activation. Additionally, as opposed to NK-92 cells, YTS cells are independent of exogenous IL-2, therefore much more locally and timely flexible while using within the patient’s body. The cells are patient independent and can therefore be used as an “off-the-shelf” therapy and does not require any patient matching since it will not cause GvHD (similar to other tested NK cell therapies^45^). Furthermore, as opposed to primary NK cells, the YTS cells are very efficiently thawed and cultivated, giving them a big advantage when being distributed to patients.

To potently work as a cancer therapy in the clinics, these cells have to be further tested with more cancer cell lines and to be introduced to good manufacturing practice (GMP)-adherent growth conditions to be able to be applied in patients. GMP adherence ensures a safe and consistent product for medical application in human patients. After doing so, these cells have the great potential to become a new therapy approach and open the door for the usage of lncRNAs as modification tools.

### Limitations of the study

The selectively enhanced cells are so far only tested on immunodeficient mice and are not yet tested in immunocompetent individuals. Additionally, the cells are not tested under GMP-compatible standards and need to be adapted to those before a possible translation into humans and the clinic.

## STAR★Methods

### Key resources table

**Table:** 

REAGENT or RESOURCE	SOURCE	IDENTIFIER
**Antibodies**		

Anti CD48 antibodies	Biolegend	BLG-336707; RRID: AB_2075176
Purified anti-IFN-γ antibody	Biolegend	BLG-502402; RRID: AB_315223
Biotinylated IFN-γ antibody	Biolegend	BLG-502504; RRID: AB_315229
Streptavidin HRP	Jackson immuno research	016-030-084; RRID: AB_2337238

**Chemicals, peptides, and recombinant proteins**		

Thiazolyl blue tetrazolium bromide (MTT)	Sigma	M5655
Calcein AM	Thermo Fisher	C1413

**Experimental models: Cell lines**		

721.221	In House	LCL 721.221 (RRID:CVCL_6263)
K562	In House	K-562 (RRID:CVCL_0004)
BCBL1	In House	BCBL-1 (RRID:CVCL_0165)
YTS	In House	YTS-Eco (RRID:CVCL_EG36)
P815	In House	P815 (RRID:CVCL_2154)
BJAB	In House	BJAB (RRID:CVCL_5711)
BW	In House	BW5147 (RRID:CVCL_3896)
8866	In House	RPMI-8866 (RRID:CVCL_1668)
EL-4	In House	EL4 (RRID:CVCL_0255)
NK-92	In House	NK-92 (RRID:CVCL_2142)
293T	In House	HEK293T (RRID:CVCL_0063)

**Experimental models: Organisms/strains**		

SCID beige mice	Envigo	CB-17/IcrHsd-Prkdc scid Lyst bg-J

**Oligonucleotides**		

NeST: FWD: GAACTAGCACAAGAGGAGTTTG	Merck	Custom DNA Oligos
NeST: REV: CTGCATGAGGAATGAGCTTT	Merck	Custom DNA Oligos
IFNG: FWD:CTTTTCAGCTCTGCATCGTT	Merck	Custom DNA Oligos
IFNG: REV: GCTACATCTGAATGACCTGCAT	Merck	Custom DNA Oligos
GAPDH: FWD: TGCCTCCTGCACCACCAACTG	Merck	Custom DNA Oligos
GAPDH: REV: CGCCTGCTTCACCACCTTCTT	Merck	Custom DNA Oligos

**Recombinant DNA**		

pHAGE-DsRED(−)eGFP(+) + NeST	Twist Bioscience	Clonal Genes Product Insert: GenBank: MK296539

### Resource availability

#### Lead contact

Prof. Ofer Mandelboim: oferm@ekmd.huji.ac.il.

#### Materials availability

There are restrictions to the availability of the plasmid and the generated cells due to ongoing patent filing.

### Experimental model and study participant details

Female SCID beige mice, an immunodeficient mouse line lacking T and B cells, showing reduced NK cell activity, were used at age 6-8 weeks. These mice belong to a congenic albino line that express both, the SCID (Prkdscid) and beige (Lystbg) mutations that are directly involved in the development of the lymphoid cells in the mice through intercross of both individual mutations in C.B-17 and C57BL/6 mice. The mice were kept at the animal facility of the Hebrew University Medical School (Ein-Kerem, Jerusalem) with 12/12h light/dark cycles at 22°C±2°C in approved specific pathogen free (SPF) housing in accordance with the guidelines of the ethics committee of the Hebrew University (ethic request number: MD-22-16847-5). Female mice were used in this project due to decreased tumor growth rate observed in female mice compared to male mice, allowing a longer period of observation during the experiment, and due to the establishment of this method in female mice in previous studies produced by this group. The used cells are continuously grown in the laboratory and are regularly authenticated through FACS staining for commonly known marker and tested for mycoplasma contaminations.

### Method details

#### Cell handling

K562, BCBL1, YTS, P815, Jurkat, BJAB, RAJI, BW, 8866, EL-4 and 721.221 cells were maintained in RPMI (Sigma-Aldrich) supplemented with 10% fetal calf serum (Sigma-Aldrich), 2 mM glutamine (Biological Industries (BI)), 1 mM sodium pyruvate (BI), 1× nonessential amino acids (BI), 100 U/ml penicillin (BI), 0.1 mg/ml streptomycin (BI).

For NK-92 cells the same medium and conditions were used, but 200U/ml IL-2 (PeproTech) were added to the medium.

#### Transduction

The plasmid construct pHAGE-DsRED(-)eGFP(+) was modified with the NeST gene sequence GenBank: MK296539 and then lentivirally transduced. Lentiviruses were replicated in 293T cells utilizing TransIT-LT1 (Mirus) and two plasmids, Gag-pol and pMDG following manufacturer’s instructions. After 48h of virus production, the supernatants were harvested and used for transducing the YTS cells. 200,000 cells were transduced at once through spinfection for 90 min at 1600 RPM at 25°C in a 96 U-plate. After 24h the cells were resupended in virus-free RPMI medium and cultured further.

#### RT-PCR

For verification of NeST, expression quantitative RT-PCR was conducted for NeST, IFNG and human GAPDH as housekeeping gene. Used primers: NeST:FWD: GAACTAGCACAAGAGGAGTTTG, REV: CTGCATGAGGAATGAGCTTT; IFNG: FWD:CTTTTCAGCTCTGCATCGTT, REV: GCTACATCTGAATGACCTGCAT; GAPDH: FWD: TGCCTCCTGCACCACCAACTG, REV: CGCCTGCTTCACCACCTTCTT.

The Viia7 Real-Time PCR system was used, and analysis was conducted via QuantStudio version 1.3.

#### Target staining

To verify the surface expression of CD48 on potential target cells, FACS stainings were conducted. The cells were incubated on ice for 30 min with 0.5μg of PE-conjugated anti CD48 antibodies (Biolegend) per 1x10^5^ cells in 100μl FACS medium. For the antibody corresponding isotype control was used. After two washing steps the stained cells were strained through a mesh and the fluorescence measured by the Cytoflex Flow Cytometer and analyzed by FACSexpress Version 6.

#### Activation assay

To assess the activation capacity of the YTS cells with the stimulus of CD48 positive (721.221, BCBL1) and negative (K562) target cells, YTS cells were incubated for 48h at 1:1 and 1:0.5 ratios with 50,000 effector cells at 37°C. The cell-free supernatant was used for IFN-γ specific sandwich ELISA. Nunc MaxiSorp™ flat-bottom ELISA plates (Invitrogen) were coated with 1μg/ml purified anti-IFN-γ (BLG-502402, Biolegend) in 50μl PBSx1 and incubated for 2h at 37°C followed by blocking with 200μl 1% BSA in PBSx1 incubated for 2h at room temperature (RT). Washing buffer of PBSx1 + 0.05% Tween-20, was used for washing the wells 3 times. 100μl of supernatant were incubated within the coated wells at 4°C overnight. The biotinylated IFN-γ detection antibody (BLG-502504, Biolegend) was then added at 1μg/ml in 100μl 1% BSA in PBSx1 and incubated for 1h at RT. Finally, streptavidin HRP (016-030-084, Jackson immuno research) 1μg/ml in 100 μl PBSx1 + 0.05% Tween-20 + 1% BSA was incubated for 30 min at RT, and quantification was performed with TMB one component substrate (Southern Biotech).

#### Killing assay

For assessment of functional killing of seYTS cells calcein-indicated killing assays were employed. For the assay BJAB and 721.221 cells were incubated with Calcein AM (1mg/ml, C1413 Thermo Fisher) in a 1:200 dilution in RPMI plus supplements for 30min at 37°C without light exposure. The target cells were washed with RPMI 2 times and distributed in 1x10^4^ cells per well in quadruplicates. The indicated effector cells were counted and incubated with the target cells at E:T ratios indicated in the figure legends. The cells were incubated for 4h and after centrifugation at 1600 RPM for 5min, the supernatant was collected and transferred into black 96 FLAT bottom plates (Greiner) for measurement in the Tecan plate reader at the calcein associated wavelength. The resulting fluorescence intensities were calculated with the spontaneous cell death and maximum cells death controls for the target cells. The spontaneous controls were incubated with medium only, the maximum cell death control was incubated with 1% triton X-100 (): ((calcein reading − spontaneous release)/(maximal release − spontaneous release))∗100 = specific lysis.

#### MTT assay

To assess the proliferation of the irradiated YTS cells, thiazolyl blue tetrazolium bromide (MTT, M5655 Sigma) was used. 50mg MTT was solubilized in 10 ml PBSx1 and sterile filtered. For each condition 2.5x10^4^ cells per well were seeded at day 0 in quadruplicates at different amounts of irradiation. After 24h intervals the cells were incubated with 10 μl of MTT for 3h in 37°C. Following the incubation, cells were resuspended in 100 μl DMSO to lyse the stained cells. After cell lysis, the colorimetric changes were measured at 590 nm.

#### Irradiation

Immune cells were irradiated at 1,000, 2,000, 3,000 and 6,000cGy utilizing the X-RAD 225 - Precision X-Ray.

#### Mice

All experiments were performed using 6–8 weeks severe combined immunodeficiency disease (SCID)-beige female mice. All mice were housed under Specific pathogen-free (SPF) conditions, in normal light/dark cycles at 22°C±2°C. All experiments were performed in accordance with the guidelines of the ethics committee of the Hebrew University Medical School (Ein-Kerem, Jerusalem). Every group of mice contained three females (n=3). Xenografts were generated by administering the indicated cells subcutaneously into the left flank region. The mice were monitored daily and sacrificed at any indication of illness such as bristled fur, difficult breathing, or tremor, among others. When tumors reached a maximal volume of 1500 mm3, all mice were sacrificed. No differences were observed between the various groups in their general health at baseline.

Injection of 1.5x10^7^ 721.221 cells subcutaneously (s.c.) in flank region per mouse, growth of tumors until steady and well palpable. Injection of PBS, YTS parental, seYTS and NK-92 cells in ratio 1:5 (3x10^6^) intravenously (i.v.) into the tail vein, with weekly booster. Monitoring of tumor growths until 1500 mm^3^ size. Tumors were collected after euthanasia.

#### Graphic illustrations

The graphic illustrations displayed in this paper were created with BioRender.com.

### Quantification and statistical analysis

Two-tailed ANOVA and Student’s T-tests in graphpad Prism version 9 were conducted, and the statistical significance, value of n, meaning of n and definition of center and dispersion are indicated in the figure legends. Data in the figures are represented as mean +/- SEM and significance is marked with ∗=p<0.06, ∗∗=p<0.01.

## Data Availability

• All analyzed and raw data reported in this paper will be shared by the lead contact upon request and has not been deposited online due to patent filing proceedings. • This paper does not report original code. • Any additional information required to reanalyze the data reported in this paper is available from the lead contact upon request.
